# Supplementary material for: Free PoC Testing for SARS-CoV-2 in Germany: Factors Expanding Access to Various Communities in a Medium-Sized City
Source: Int J Environ Res Public Health. 2022 Apr 13;19(8):4721. doi: 10.3390/ijerph19084721 (PMC9027734; doi:10.3390/ijerph19084721)
Supplement: Supplementary file 1 [file ijerph-19-04721-s001.zip › ijerph-1651843-supplementary.pdf]

Supplementary Materials

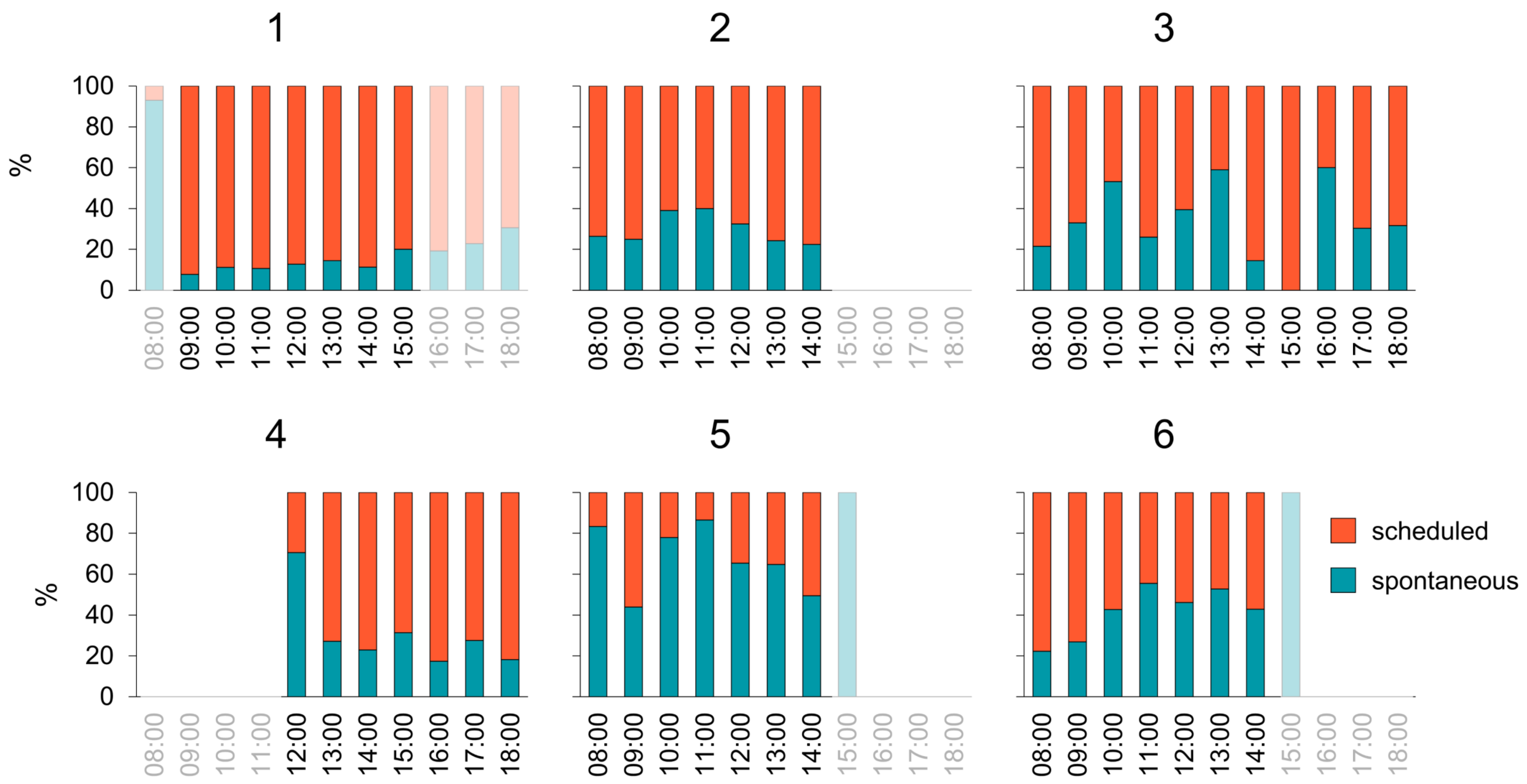

Figure S1. Scheduled versus spontaneous tests in relation to time

People with scheduled (dark grey) and spontaneous appointment (light grey) analyzed for opening hours.1. Central Bus Station, 2. Office Block, 3. Theatre, 4. District Community Center, 5. Park & Ride, 6. Suburban Test Center.

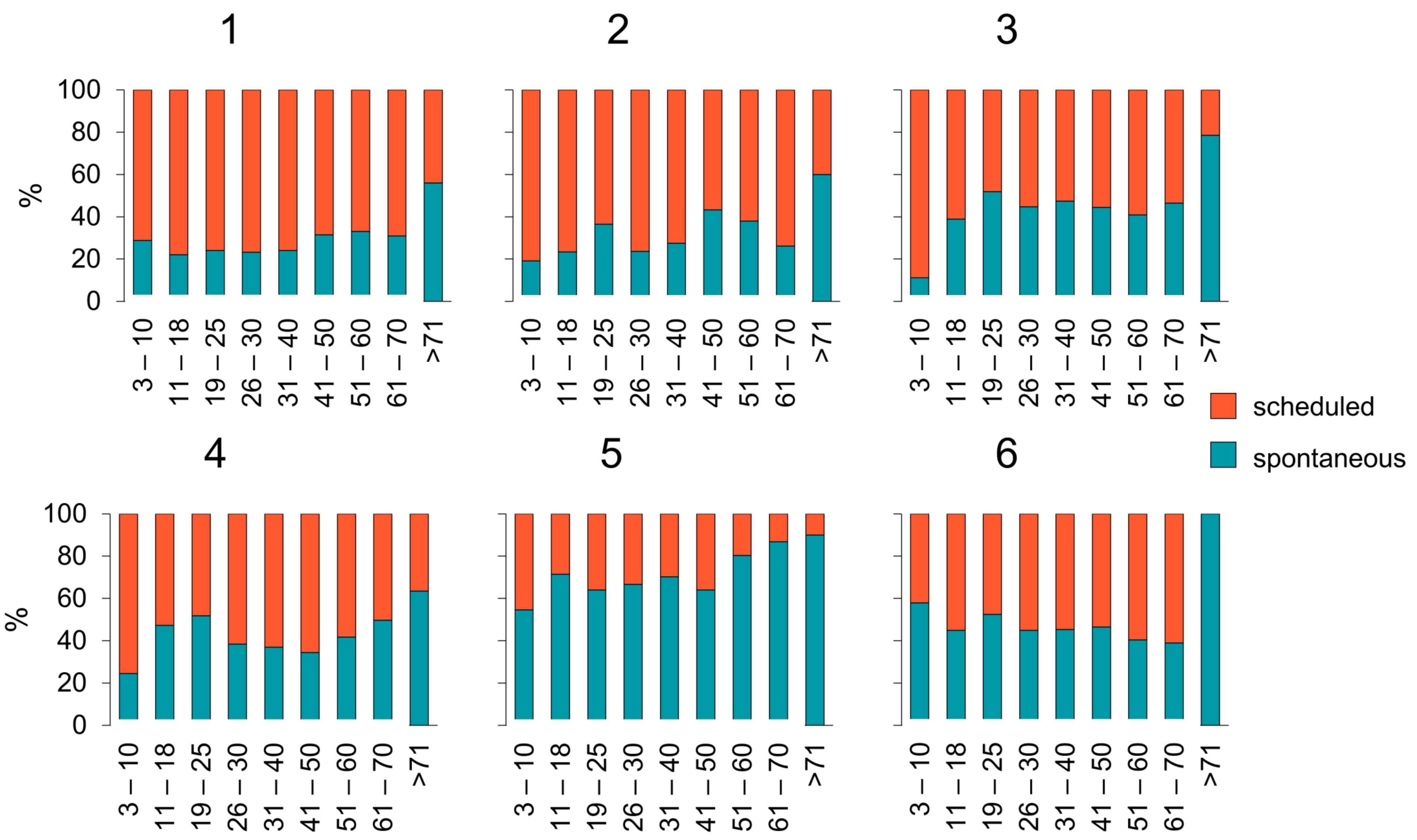

Figure S2. Scheduled versus spontaneous tests in relation to age

People with scheduled (dark grey) and spontaneous appointment (light grey) analyzed for age groups. 1. Central Bus Station, 2. Office Block, 3. Theatre, 4. District Community Center, 5. Park & Ride, 6. Suburban Test Center.

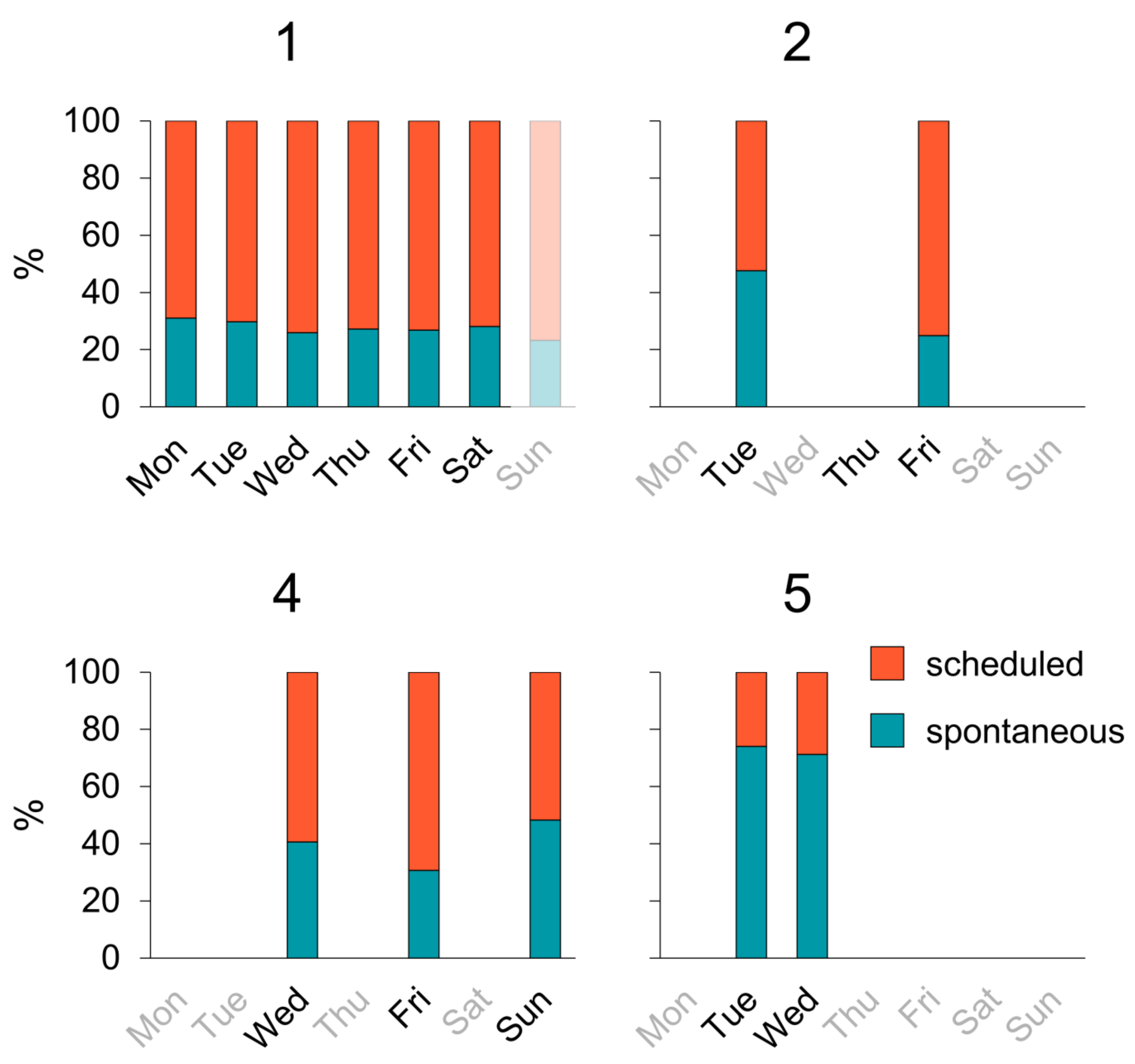

Figure S3. Scheduled versus spontaneous tests in relation to weekdays

People with scheduled (dark grey) and spontaneous appointment (light grey) analyzed for weekdays. 1. Central Bus Station, 2. Office Block, 3. Theatre, 4. District Community Center, 5. Park & Ride, 6. Suburban Test Center.

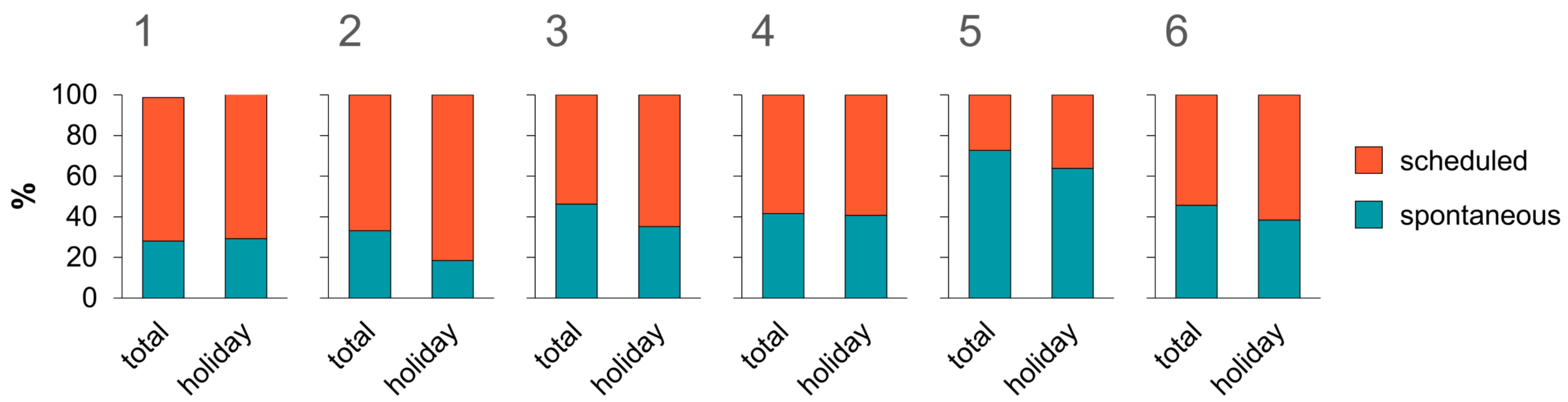

Figure S4. Scheduled versus spontaneous tests in relation to days before holidays

People with scheduled (dark grey) and spontaneous appointment (light grey) analyzed in total and for days before holiday ("holiday") 1. Central Bus Station, 2. Office Block, 3. Theatre, 4. District Community Center, 5. Park & Ride, 6. Suburban Test Center.

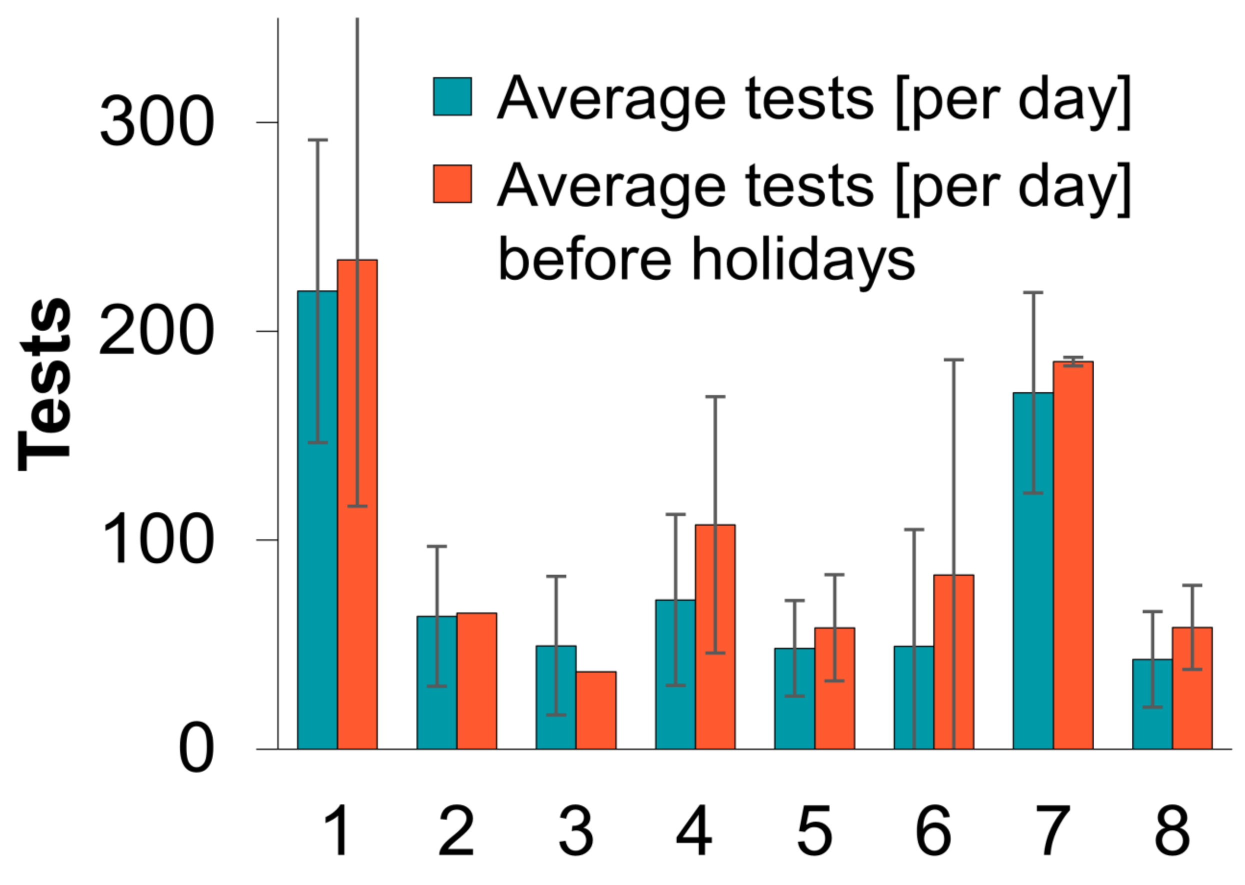

Figure S5. Average Tests per day in total and on days before holidays

Bars and error bars represent average with standard deviation. Since not all locations have the same number of opening days, the number of analyzed days is as following: (number of all open days/number of open days before holidays) 1. Bus (73/7), 2. Office Block (16/1), 3. Theatre (15/1), 4. District Community Center (20/3), 5. Park & Ride (12/2), 6. Suburban Test Center (12/3), 7. Hospital (30/2), 8. Bus (45/4).

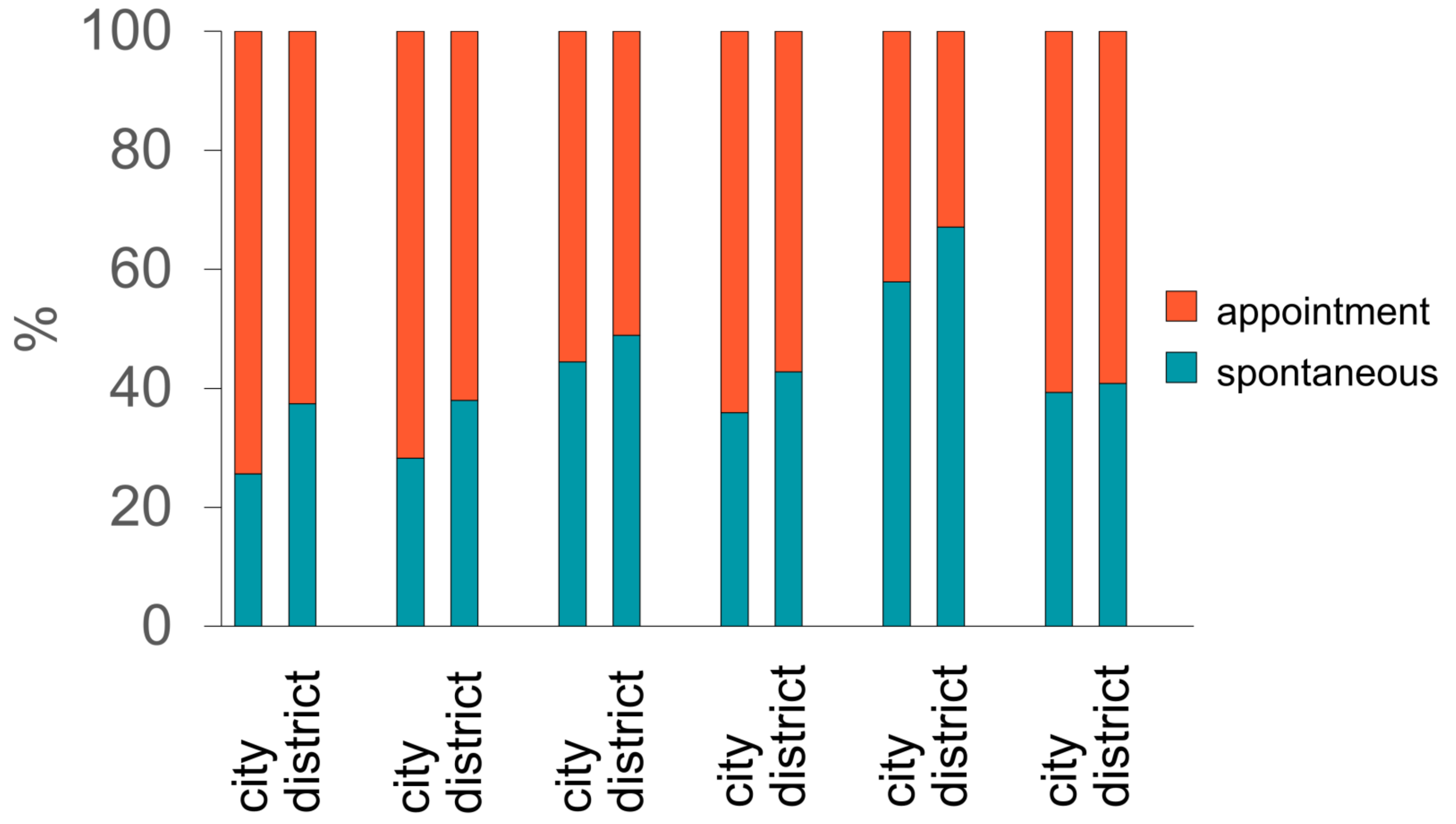

Figure S6. Scheduled versus spontaneous tests in relation to residence

People with scheduled (dark grey) and spontaneous appointment (light grey) analyzed for residence in the city and district. 1. Central Bus Station, 2. Office Block, 3. Theatre, 4. District Community Center, 5. Park & Ride, 6. Suburban Test Center.
